# Resection of a Large Growing Mediastinal Germ Cell Tumor Using a Multidisciplinary Approach

**DOI:** 10.3390/curroncol31010003

**Published:** 2023-12-21

**Authors:** Alison Greene, Lori Wood, Philip Champion, Mathieu Castonguay, Matthias Scheffler, Catherine Deshaies, Jeremy Wood, Daniel French

**Affiliations:** 1 Division of Cardiac Surgery, Department of Surgery, Dalhousie University, Queen Elizabeth II Hospital, Halifax, NS B3H 2Y9, Canada; d54amg@mun.ca (A.G.);; 2 Division of Medical Oncology, Department of Medicine, Dalhousie University, Queen Elizabeth II Hospital, Halifax, NS B3H 2Y9, Canada; 3 Division of Medical Oncology, Department of Medicine, Dalhousie University, Queen Elizabeth Hospital, Charlottetown, PEI C1A 8T5, Canada; pechampion@ihis.org; 4 Department of Pathology, Dalhousie University, Queen Elizabeth II Hospital, Halifax, NS B3H 2Y9, Canada; mathieu.castonguay@nshealth.ca; 5 Division of Cardiac Anesthesia, Department of Anesthesia, Pain Management and Perioperative Medicine, Dalhousie University, Queen Elizabeth II Hospital, Halifax, NS B3H 2Y9, Canada; 6 Division of Thoracic Surgery, Department of Surgery, Dalhousie University, Queen Elizabeth II Hospital, Halifax, NS B3H 2Y9, Canada

**Keywords:** germ cell tumor, rhabdomyosarcoma, transsternal resection

## Abstract

Mediastinal germ cell tumors (GCTs) are rare. Post-chemotherapy residual masses in patients with a nonseminomatous GCT require resection. A patient with a large mediastinal GCT involving the left subclavian artery, superior vena cava (SVC) and hilum of the right lung is presented. Despite a biochemical response to chemotherapy, the tumor enlarged on serial imaging. With guidance from medical oncology, a multidisciplinary surgical team, including cardiac anesthesia, cardiac surgery and thoracic surgery resected the tumor with a staged reconstruction of the SVC. The procedure was well tolerated and yielded clear margins. The final pathology showed a significant associated component of rhabdomyosarcoma.

## 1. Introduction

Extragonadal germ cell tumors (GCTs) are rare, comprising only 2–5.7% of all GCTs [1]. Given the rarity of these tumors, specific treatment strategies remain ill-defined and these tumors continue to carry a poor prognosis [2]. Furthermore, GCTs with malignant somatic transformations have been well described [3]. This uncommon variant should be considered when a GCT grows on systemic therapy. This case highlights the importance of multidisciplinary and national expert collaboration in managing these complex and challenging tumors. Written consent was obtained from the patient to publish this case report in compliance with the local institutional research ethics board requirements.

## 2. Case

**Figure 1:**
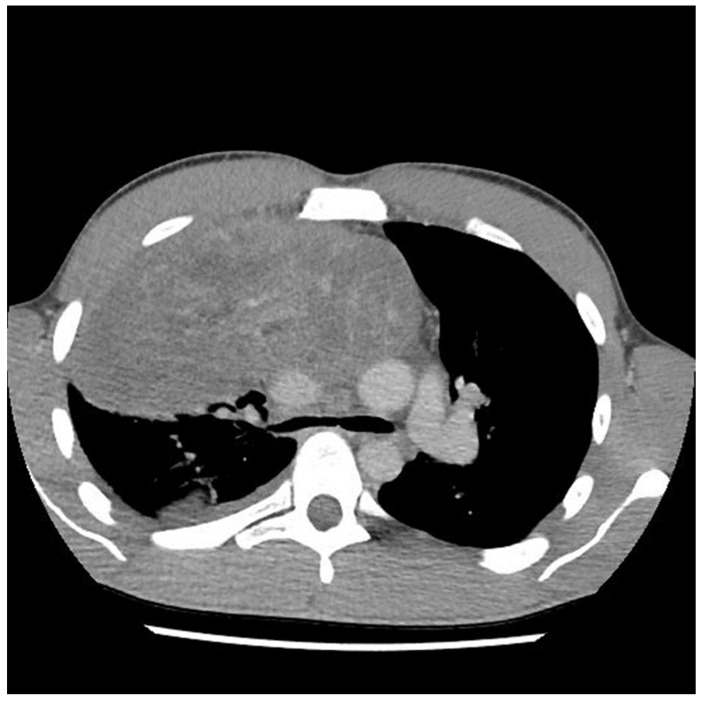
Computed tomography (CT) scan image of mediastinal mass at the time of initial presentation.

A 29-year-old male with no previous medical history presented to the emergency department in May 2021 with symptoms of acute appendicitis. He was ultimately found to have a large mediastinal mass (Figure 1). A needle biopsy indicated predominantly mature teratomas with some elements of a yolk sac tumor (YST). Biochemical workup revealed an initial alpha fetal protein (AFP) level of 1654.51 ng/mL, and the beta human chorionic gonadotropin (β-HCG) level was slightly elevated at 7.2 (IU/L).

**Figure 2:**
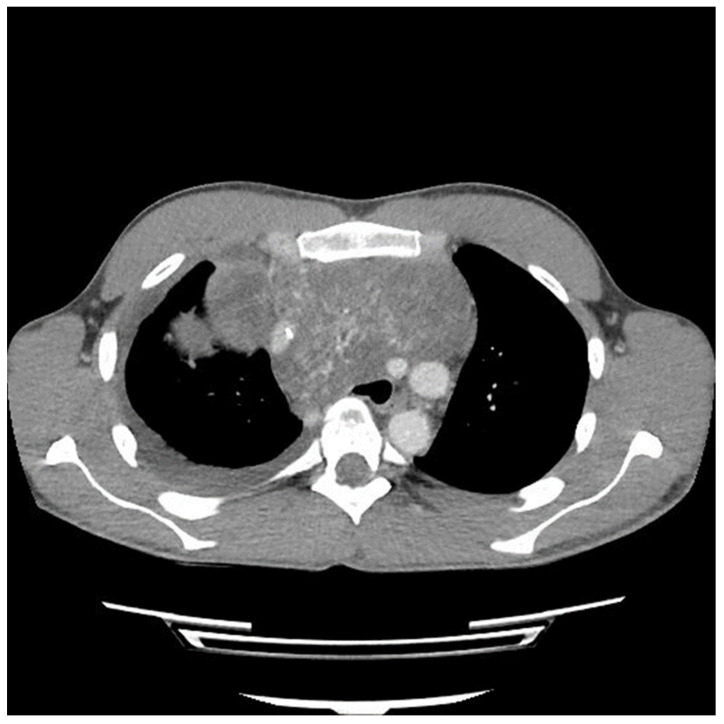
Computed tomography (CT) scan image of mediastinal mass after two cycles of chemotherapy showing interval growth of tumor despite a biochemical response.

Etoposide, ifosfamide and cisplatin (VIP) chemotherapy was initiated. Bleomycin was not used to avoid pulmonary toxicity, anticipating surgical resection. After the first and second VIP cycles, AFP levels decreased to 1326.96 ng/mL and 219.57 ng/mL, respectively. However, a scheduled computed tomography (CT) scan showed an enlargement of the mass (Figure 2).

The discrepancy between imaging and biochemical responses was evaluated by the patient’s medical oncology team, along with members of a virtual Canadian GCT Discussion Group via email correspondence. Given that any further potential growth could make him unresectable and that the initial pathology showed a significant component of teratoma, which is not chemotherapy-responsive, the consensus was to proceed with surgical resection. During the discussion period and perioperative work-up period, a third cycle of VIP was completed prior to resection. AFP normalized prior to resection.

Four months after initiating chemotherapy, a multidisciplinary team—consisting of cardiac anesthetists, cardiac surgeons and thoracic surgeons—proceeded with en bloc resection of the residual tumor.

Preoperatively, the patient demonstrated that he could lay supine for 20 min without hemodynamic consequences. Following anesthesia induction, bronchoscopy revealed occlusion of the right mainstem bronchus, precluding the placement of a double-lumen tube. The rapid development of thoracic compartment syndrome then only allowed for the placement of a 6.5-mm single-lumen endotracheal tube. While hypoxia did not occur, only minimal CO_2_ return was noted until the chest was opened. In anticipation of SVC syndrome, an additional large central line was placed in the left femoral vein.

**Figure 3:**
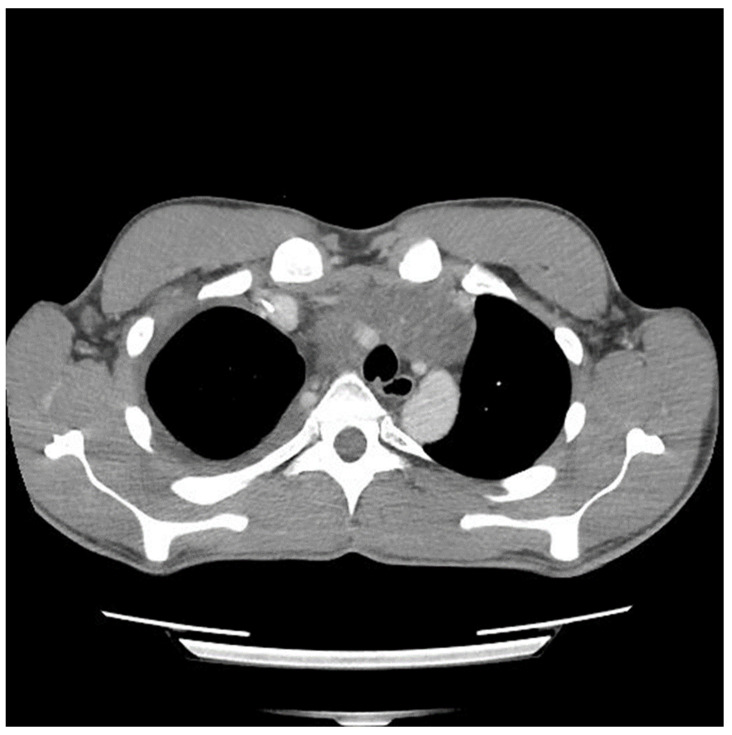
Computed tomography (CT) scan image of mediastinal mass after two cycles of chemotherapy showing encasement of left subclavian artery.

A sternotomy was performed using an oscillating saw. A plane was developed between the pericardial fat and pericardium at the level of the inferior vena cava and carried towards the left side of the tumor. Entering the left pleural space allowed release of the tumor from its left lateral attachments and identification of the left phrenic nerve. Fortunately, despite the preoperative CT (Figure 2 and Figure 3) suggesting encasement of the left subclavian artery, distracting the tumor to the right revealed it could be unwrapped and peeled off this vessel, allowing perseveration of it.

On the right side, the tumor was densely adherent to the lung. A fourth intercostal space trapdoor in the right anterior thoracotomy improved exposure. Despite concerning preoperative images, the tumor was not adherent to the hilum of the right lung. An en bloc wedge resection of the right lung released the tumor from the right pleural space.

With the tumor freed on the left and right sides, it only remained attached to a large vessel, which was totally encased. The patient’s cardiac anatomy had been significantly distorted by tumor growth, making structures difficult to reliably identify. Additionally, the rotation of the tumor compressed the right heart, resulting in hemodynamic instability. The intraoperative transesophageal echocardiogram (TEE) showed the superior vena cava (SVC) coursing inferior to the tumor. Based on this finding, the vessel was ultimately ligated, allowing the removal of the tumor.

The total operating theater time was 12 h and 57 min. The duration of the operation was 9 h and 11 min. During the case, significant bleeding and coagulopathy ensued, necessitating the transfusion of 23 units of packed red blood cells, 6.8 L of fresh frozen plasma, three adult bags (12 units) of platelets, and 10 g of fibrinogen concentrate. Hemodynamic instability was aggressively managed with norepinephrine, vasopressin and epinephrine infusions, thereby avoiding cardiopulmonary bypass.

On postoperative days (POD) 1 and 2, the patient developed progressive swelling of his upper extremities concerning SVC syndrome. On POD 3, a CT scan with upper body venous contrast confirmed amputation of the SVC from the confluence of the subclavian and internal jugular veins to 3 cm above the right atrial junction with nonocclusive thrombus in large tributaries and the development of collateralization. The patient was taken back to the operating room for SVC reconstruction using a bovine pericardial tube.

On POD 6, the patient was extubated. At that point, left mydriasis and exotropia were obvious. Visual acuity was preserved on testing. These findings were consistent with a left afferent pupillary defect secondary to ischemia, likely the result of a transient increase in intracranial pressure. On POD 13, the patient developed a *Bacillus cereus* central line infection, which was treated with vancomycin. He was discharged home on POD 25 and returned to work as a lobster fisherman 6 months later. Fortunately, the patient’s ocular symptoms completely resolved after 1 year.

**Figure 4:**
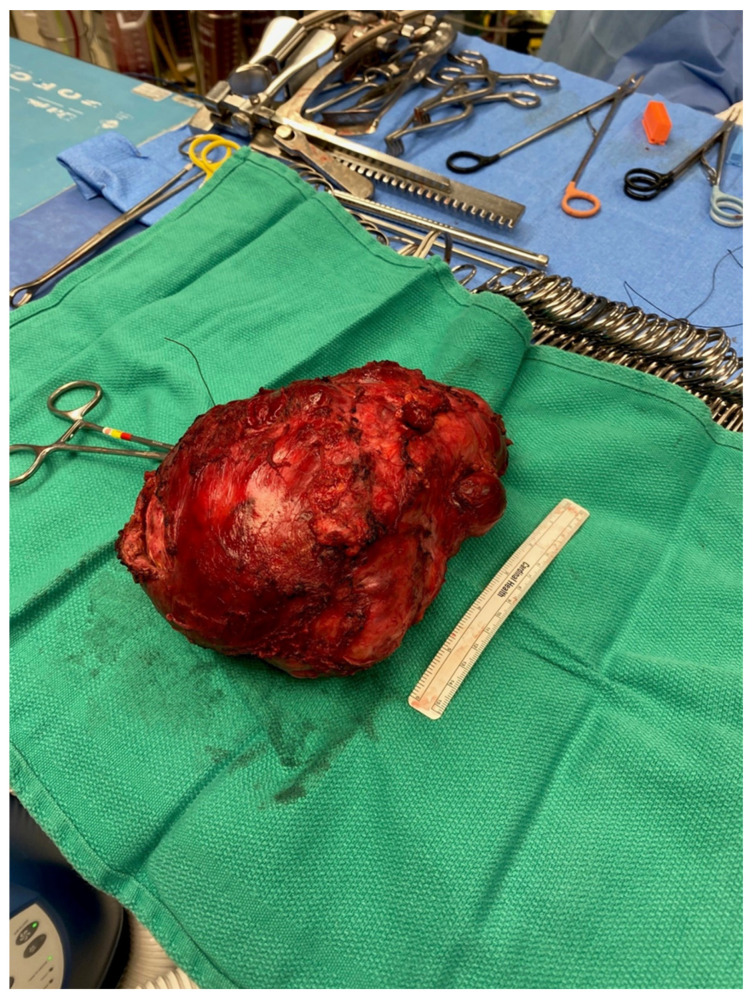
A photograph of the resected specimen.

The resected tumor measured 21.0 × 17.0 × 12.0 cm and weighed 1.5 kg, and its histopathological evaluation revealed a mixed germ cell tumor (approximately 95% mature teratoma and 5% yolk sac tumor) associated with somatic-type solid malignancy in the form of rhabdomyosarcoma, forming multiple expansile regions throughout the tumor (Figure 4). The teratoma included bronchial mucosa (with cartilage, smooth muscle, fat, myxoid stroma and seromucous glands), glands resembling pancreaticobiliary epithelium, and cysts lined by squamous epithelium. Immunohistochemical studies revealed reactivity of these neoplastic cells to antibodies directed against CK AE1/AE3 and p40 (focal), but not to those directed against SALL4, AFP, glypican, OCT4 or GATA3. The yolk sac tumor component grew in glands, microcysts and solid patterns, with focal parietal differentiation and areas of prominent cellular pleomorphism. Immunohistochemical studies reveal reactivity of these neoplastic cells to antibodies directed against CK AE1/AE3, AFP, glypican, SALL4, OCT4 and GATA3, but not to those directed against HCG or CD30. The stroma included multiple expansile regions of rhadbomyosarcomatous differentiation, scattered throughout the tumor. The malignant skeletal muscle cells varied in differentiation, including poorly differentiated pleomorphic and spindle malignant cells, and reactivity to antibodies directed against desman and S100 protein but not to those directed against alpha-actin, MYF or SOX10. Approximately 10% of the tumor was necrotic. There was invasion of intratumoral veins (evaluated with elastic stains). While most of the tumor appeared marginally resected, the tumor focally involved the inked right and anterior soft tissue margins. The tumor was adherent to the adventitia of the superior vena cava (without medial invasion) and invaded the right lung (evaluated with elastic stains). There was thymic tissue around the tumor, without significant histopathologic abnormalities.

Discussion with local experts and the national GCT discussion group occurred regarding further chemotherapy for the residual YST or adjuvant chemotherapy or radiation therapy for the rhabdomyosarcoma; however, a decision was made to initiate close surveillance with a low threshold to intervene in response to any radiographic evidence of recurrent disease. CT scans, tumor markers and clinic visits were scheduled every 3–6 months for 3 years and then annually. At 1 year, there was no evidence of recurrent disease.

## 3. Discussion

This case highlights the importance of a multidisciplinary approach to mediastinal GCTs.

Advanced GCTs are typically treated with upfront systemic therapy and routine reassessment using biochemistry and cross-sectional imaging. The surgical team must assess the respectability of the tumor, paying particular attention to vital structures, along with the patient’s ability to tolerate the resection. However, the medical oncology team plays a vital role in determining the appropriateness and timing of post-chemotherapy residual mass resection. In this case, the patient was young, active and fit, allowing them to tolerate resection. Based on imaging, the mass was encasing and abutting critical structures, making its success uncertain. Nevertheless, tumor growth on systemic therapy made resection his only option for survival [4,5].

The preoperative CT (Figure 1, Figure 2 and Figure 3) identified the involvement of three critical structures: (1) left subclavian artery, (2) right hilum of the lung and (3) SVC. Interestingly, only the encasement of the SVC was confirmed at the time of the surgery. The tumor enveloped the left subclavian on either side but could be peeled off, allowing preservation of the vessel. The tumor abutted the right hilum of the lung but maintained a potential space, so the lesion could simply be lifted off this region. The mass was densely adherent to the lung parenchyma, which was released with cautery. This resulted in a postoperative air leak, which was resolved with conservative management. The encasement of the SVC was the greatest operative challenge. Because the tumor significantly distorted the anatomy of the heart, the SVC was misidentified at the time of surgery, both by direct inspection and TEE evaluation. In retrospect, the use of adjunct techniques, such as a bubble study or the introduction of a wire under direct visualization, could have been helpful in ascertaining the structure intraoperatively. Transaction would have still been required for en bloc resection, but immediate reconstruction of the vessel would have then been possible.

Other authors have had similar experiences with challenging dissections. Hayati et al. performed a piecemeal resection of 3.5 kg of GCT in a 16-year-old male through a posterolateral thoracotomy. In this case, only a teratomatous component of the tumor was found in the final pathology [6]. Fritzcshe et al. removed a 3-kg GCT via a right pneumonectomy, revealing a mixture of an immature teratoma on final pathology [7]. In the case presented here, a sternotomy was extended to a right anterior thoracotomy to allow exposure and dissection of the right hilum and right-sided pericardial structures. It is important to study preoperative imaging to consider the optimal approach to facilitate dissection and resection of these large, growing tumors.

Interestingly, the patient was able to tolerate laying supine for 20 min prior to surgery without signs of respiratory or cardiovascular compromise. The patient’s baseline fitness likely blunted the impact that the tumor had on his cardiac output and airway. However, the mass effect became apparent with anesthesia.

**Figure 5:**
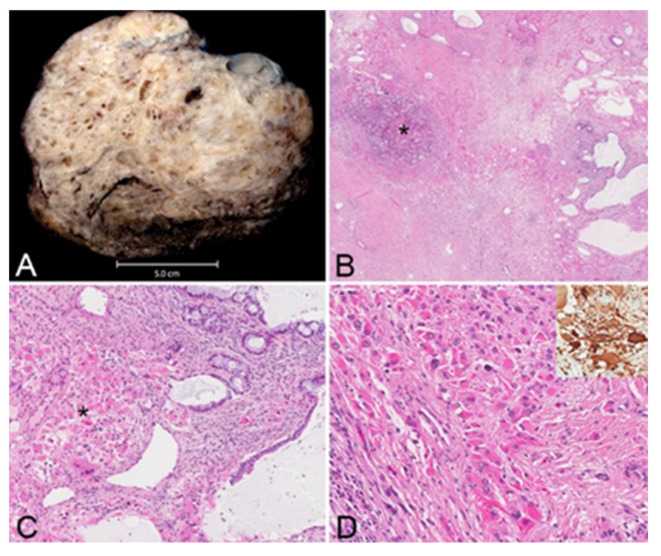
(**A**), Cross-section photograph of resected tumor specimen; (**B**) low-power photomicrograph, with mature teratoma (right side of image) and yolk sac tumor (*) components, accompanied by fibrosis (H&E, original magnification ×20); (**C**) rhabdomyosarcoma (*) admixed with mature teratoma (right side of image) (H&E, original magnification ×100); (**D**) rhabdomyosarcoma cells (H&E, original magnification ×200) (inset, desman immunohistochemical study).

The final pathology revealed a component of rhabdomyosarcoma (i.e., a somatic-type solid malignancy) in a mixed germ cell tumor otherwise comprised largely of mature teratoma (see Figure 5). This is an uncommon but well-documented occurrence, which appears to portend a less favorable prognosis [8]. While various somatic types of malignancy have been described in association with GCTs, rhabdomyosarcoma is the most common type in mediastinal GCTs, and their reduced chemosensitivity likely explains their persistence following chemotherapy [8]. A review by multiple local and national tumor boards consistently suggested close surveillance over chemotherapy or radiation.

In summary, this case highlights the complexity of the management of mediastinal GCTs. Despite unfavorable preoperative imaging, assembling a multidisciplinary team capable of managing the mass effect of mediastinal tumors and reconstructing critical vessels can allow for the resection of these tumors.

## Data Availability

Data is contained within the article.
